# A multiphase contrast-enhanced CT radiomics model for prediction of human epidermal growth factor receptor 2 status in advanced gastric cancer

**DOI:** 10.3389/fgene.2022.968027

**Published:** 2022-10-07

**Authors:** Tingting Ma, Jingli Cui, Lingwei Wang, Hui Li, Zhaoxiang Ye, Xujie Gao

**Affiliations:** ^1^ Department of Radiology, Tianjin Cancer Hospital Airport Hospital, Tianjin, China; ^2^ Department of Radiology, Tianjin Medical University Cancer Institute and Hospital, Tianjin, China; ^3^ National Clinical Research Center for Cancer, Tianjin, China; ^4^ Tianjin’s Clinical Research Center for Cancer, Tianjin, China; ^5^ Department of Bone and Soft Tissue Tumor, Tianjin Medical University Cancer Institute and Hospital, Tianjin, China; ^6^ Department of General Surgery, Weifang People’s Hospital, Weifang, Shandong, China; ^7^ The Key Laboratory of Cancer Prevention and Therapy, Tianjin, China; ^8^ Department of Gastrointestinal Cancer Biology, Tianjin Medical University Cancer Institute and Hospital, Tianjin, China; ^9^ Key Laboratory of Cancer Immunology and Biotherapy, Tianjin, China

**Keywords:** radiomics, advanced gastric cancer, HER2, computed tomgraphy, nomograph

## Abstract

**Background:** Accurate evaluation of human epidermal growth factor receptor 2 (HER2) status is of great importance for appropriate management of advanced gastric cancer (AGC) patients. This study aims to develop and validate a CT-based radiomics model for prediction of HER2 overexpression in AGC.

**Materials and Methods:** Seven hundred and forty-five consecutive AGC patients (median age, 59 years; interquartile range, 52–66 years; 515 male and 230 female) were enrolled and separated into training set (*n* = 521) and testing set (*n* = 224) in this retrospective study. Radiomics features were extracted from three phases images of contrast-enhanced CT scans. A radiomics signature was built based on highly reproducible features using the least absolute shrinkage and selection operator method. Univariable and multivariable logistical regression analysis were used to establish predictive model with independent risk factors of HER2 overexpression. The predictive performance of radiomics model was assessed in the training and testing sets.

**Results:** The positive rate of HER2 was 15.9% and 13.8% in the training set and testing set, respectively. The positive rate of HER2 in intestinal-type GC was significantly higher than that in diffuse-type GC. The radiomics signature comprised eight robust features demonstrated good discrimination ability for HER2 overexpression in the training set (AUC = 0.84) and the testing set (AUC = 0.78). A radiomics-based model that incorporated radiomics signature and pathological type showed good discrimination and calibration in the training (AUC = 0.85) and testing (AUC = 0.84) sets.

**Conclusion:** The proposed radiomics model showed favorable accuracy for prediction of HER2 overexpression in AGC.

## Introduction

Gastric cancer (GC) is the fifth common malignancy and the fourth reason of cancer-related death (Sung et al., 2021). In addition, GC is the most common and fatal cancer in men in several South-Central Asian countries (Sung et al., 2021). Attributed to the endoscopic screening, the mortality of GC decreased in some courtiers. However, given low screening rates in China, majority of the GC patients are identified at advanced stage with poor prognosis. Human epidermal growth factor receptor 2 (HER2) is a crucial therapeutic target for various types of solid tumors, including GC. The overexpression of HER2 associates with an unfavorable prognosis and plays a crucial role of tumorigenesis in GC (Wang et al., 2018; Luo et al., 2019). Trastuzumab, a monoclonal antibody targeting HER2, can inhibit tumor cell proliferation through blocking downstream signal transduction pathway. The ToGA (Trastuzumab for Gastric Cancer) trail demonstrated that anti-HER2 monoclonal antibody Trastuzumab with chemotherapy prolonged survival of HER2 positive advanced gastric cancer (AGC) patients (Bang et al., 2010). For AGC patients with HER2 overexpression, Trastuzumab is recommended as the first-line target therapy by National Comprehensive Cancer Network (NCCN) guidelines. However, GC is a highly heterogenous disease (Nishida et al., 2015; Wakatsuki et al., 2018). Although multi-regional sampling was used to determine HER2 status, the histological examination of surgical resection or biopsy samples only reveals a fraction of tumors. Thus, conventional HER2 testing for GC patients still has a high risk of false negative of HER2 overexpression. Patients with incorrect test results may lose the opportunity of targeted therapy. Therefore, an accurate evaluation of HER2 status is of particular significance for GC patients.

AI techniques provide new methods to process images and translate them into quantitative data, allowing the identification of microscopic features of tumor that invisible by human eyes (Bi et al., 2019). Accumulating evidence shows that radiomics can be applied in various aspects including diagnosis, prediction of metastasis risk, survival and treatment response for GC patients (Ma et al., 2017; Li et al., 2019). Previous studies have reported that gene mutation status could also be predicted by radiomics features (Zhao et al., 2019; Cui et al., 2020). Besides, radiomics analysis combined with 3 dimensions (3D) reconstruction technology allows the extraction of image features from the whole volume of the lesions, providing more comprehensive information of intratumor heterogeneity (Bi et al., 2019). Therefore, the radiomics method can serve as a robust and noninvasive biomarker in the evaluation of tumor gene expression. Nevertheless, few studies have explored the clinical application potential of radiomics method for evaluation of HER2 status in AGC (Wang et al., 2021).

Therefore, in the current study, we established and validated a multiphase contrast-enhanced CT (CECT)-based radiomics model for predicting HER2 overexpression in AGC patients.

## Materials and methods

### Study population

Seven hundred and forty-five consecutive AGC patients including 515 males (median age, 61 years; interquartile range, 54–66 years) and 230 females (median age, 57 years; interquartile range, 49–64 years) were consecutively enrolled from July 2015 to December 2017. According to the time of diagnosis, all patients were separated into training (*n* = 521) and testing set (*n* = 224) at a ratio of 7:3. The inclusion criteria: (Sung et al., 2021) patients received radical gastrectomy or endoscopic biopsy; (Wang et al., 2018) AGC diagnosis was confirmed pathologically; (Luo et al., 2019) abdominal CECT scans were performed within 2 weeks before biopsy or surgery; (Bang et al., 2010) HER2 status was available; (Nishida et al., 2015) Imaging quality meet the requirements of analysis: 1) gastric cavity was sufficiently distend; 2) images without sever peristaltic and respiratory artifacts. The exclusion criteria: (Sung et al., 2021) lack of complete clinical records; (Wang et al., 2018) patients received any treatment prior to CT scan; (Luo et al., 2019) patients suffered from other malignant disease.

This retrospective was conducted under the approval of our institutional ethical review board. The requirement for informed consent was waived.

The clinical and pathological information of all patients were obtained. Pathologic staging was determined in accordance to the eighth edition AJCC staging system. The patient’s recruitment flow chat is illustrated in the Supplementary Figure S1.

### Human epidermal growth factor receptor 2 status assessment

The HER2 status was assessed according to NCCN guidelines (Ajani et al., 2022). HER2 testing was performed as previously descripted (Bang et al., 2010). Immunohistochemistry (IHC) and Fluorescence in‐situ hybridization (FISH) were used to determine HER2 status. IHC staining score of 3+ were considered positive. IHC staining score of 2+ with FISH+ were also deemed positive. Patients with IHC score of 0, 1+ or 2+ with FISH− were considered negative.

### Lesion segmentation and feature extraction

Abdominal CECT images in arterial phase (AP), portal phase (PP) and delay phase (DP) were analyzed. 3D Slicer software was used for constructing volume of interest (VOI) of lesions. The VOIs of each lesion of three phases were manually delineated along the margin of tumor. The adjacent fluid or air were carefully avoided into contours and reconstructed sagittal and coronal images were used as reference. The procession of the segmentation was done under the consensus of 2 radiologists (XJG and TTM, with 7 and 10 years of interpretation experience in abdominal CT imaging). Feature extraction was implemented through utilizing an open-source platform (PyRadiomics 2.2.0) (Gao et al., 2021). A total of 859 features were extracted and grouped into four types: size and shape, first-order statistical, textural features, and wavelet features. The Details of the CT scanning protocol, image pre-processing and radiomic features are shown in the Supplementary methods and Supplementary Table S1.

### Radiomics signature establishment

**FIGURE 1:**
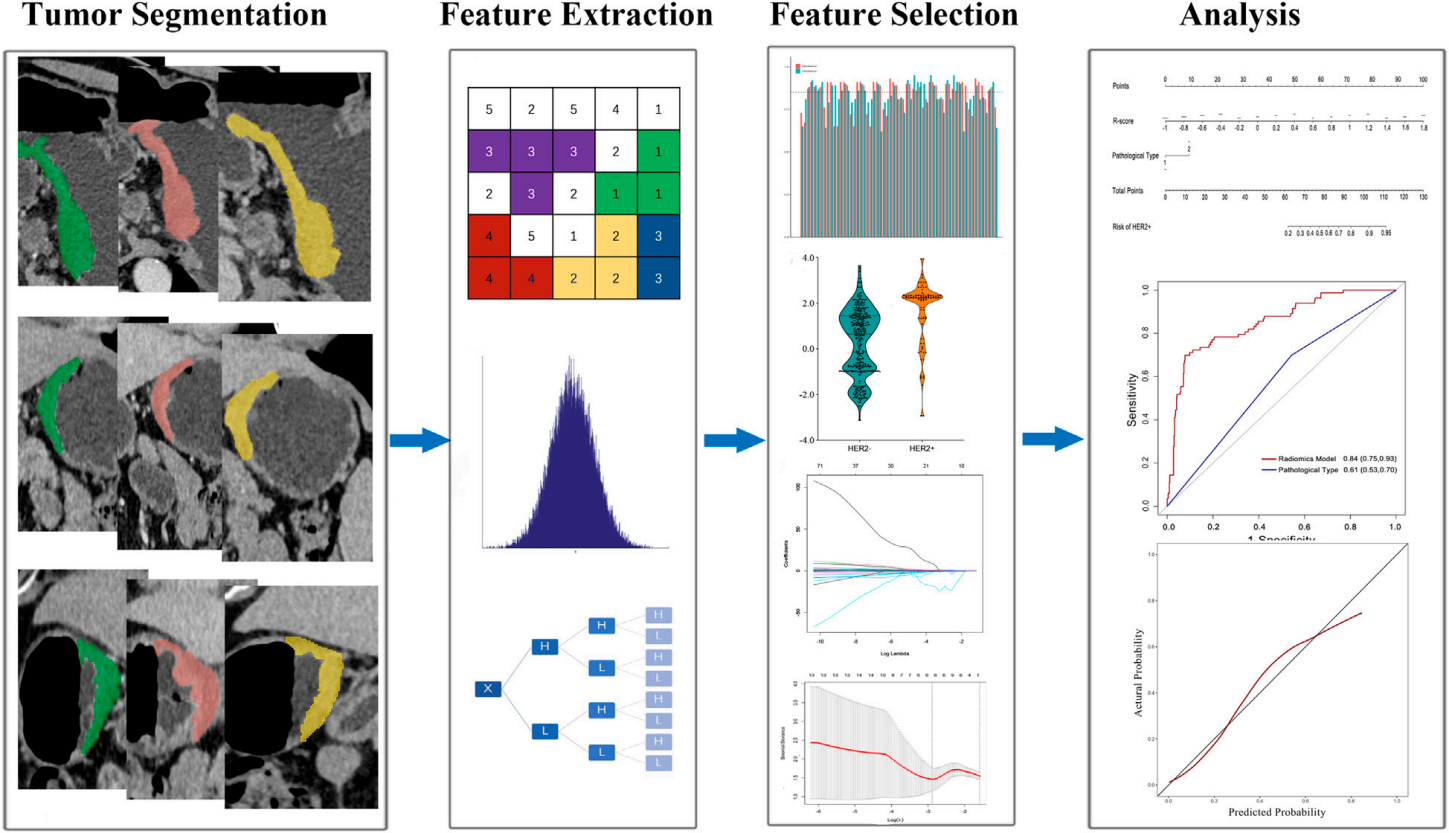
Flowchart of study design.

The flowchart of overall radiomics analysis is shown in Figure 1. In order to ensure the reliability of the selected features, we assessed the intra- and inter-observer agreement using intra- and interclass correlation coefficients. We randomly chosen 120 patients from the training set and the VOI segmentation were independently performed by 2 readers. Reader 1 repeated the procedure 2 weeks later. The features were regarded as stable if the intra- and interclass correlation coefficients values were higher than 0.85. Then, the patients were divided into HER2+ and HER2−groups. The Mann-Whitney *U* test was used to identify features that differed significantly between the 2 groups. Bonferroni correction was used to limit type 1 errors, and features with FDR adjusted *p* < 0.05 were selected for further analysis. The least absolute shrinkage and selection operator (LASSO) model was subsequently utilized to build a radiomics signature with non-zero coefficients. The 10-fold cross-testing was employed to identify the optimal regularization parameter *λ*. The radiomics scores (R-score) were calculated based on the fitting formula of the radiomics signature for all patient (Huang et al., 2016). The predicting accuracy of radiomic signature were evaluated in both sets.

### Nomogram establishment and validation

Univariable and multivariable logistic regression model were utilized to identify independent risk factors of HER2 overexpression in the training set. Then, a radiomics model was established and represented as a nomogram.

### Statistical analysis

All statistical analyses were performed using R (version 3.4.2) with packages including “glmnet,” “pROC” and “Survminer.” Differences between categorical variables were compared with chi-squared test or Fisher’s exact test. Differences between continuous variables were compared with the student’s *t*-test or the Mann-Whitney test. The Dice similarity coefficient was used for evaluation the inter-observer reproducibility of lesion segmentation. The discrimination ability of the radiomics signature was determined with the receiver operating characteristic (ROC) curve. Maximized Youden index was used to find the best cutoff threshold of R-score for classifying patients into low- and high risk of HER2 overexpression. Calibration of the nomogram was evaluated by Hosmer-Lemeshow test.

## Results

### Analysis of clinical information

The training set contained 83 (15.9%) HER2+ patients, while the testing set contained 31 (13.8%) HER2+ patients. The positive rate of HER2 in intestinal-type GC was significantly greater than that in diffuse-type GC. No significant differences were showed in HER2+ and HER2−groups in both sets in terms of age, gender, TNM stage, tumor sites and differentiation status. The clinicopathological characteristics of all patients are shown in Table 1.

**TABLE 1:** Characteristics of the study population.

Variable	Training set (*n* = 521)			Testing set (*n* = 224)		
	HER2− (*n* = 438)	HER2+ (*n* = 83)	*P*	HER2− (*n* = 193)	HER2+ (*n* = 31)	*P*
Age			0.86			0.26
<60	217 (49.5)	42 (50.6)		91 (47.2)	18 (58.1)	
≥60	221 (50.5)	41 (49.4)		102 (52.8)	13 (41.9)	
Gender			0.12			0.10
Male	306 (69.9)	60 (72.3)		127 (65.8)	22 (71.0)	
Female	132 (30.1)	23 (27.7)		66 (34.2)	9 (29.0)	
Tumor Site			0.38			0.91
Upper	135 (30.8)	27 (32.5)		71 (36.8)	11 (35.5)	
Middle	66 (15.1)	15 (18.1)		42 (21.7)	5 (16.1)	
Lower	183 (41.8)	27 (32.5)		59 (30.6)	10 (32.3)	
Overlap	54 (12.3)	14 (16.9)		21 (10.9)	5 (16.1)	
Pathologic T stage			0.69			0.53
T2	125 (28.5)	27 (32.5)		39 (20.2)	9 (29.0)	
T3	40 (9.1)	9 (10.8)		26 (13.5)	4 (12.9)	
T4	273 (62.3)	47 (56.6)		128 (66.3)	18 (58.1)	
Pathologic N stage			0.22			0.30
N0	140 (32.0)	33 (39.8)		70 (36.3)	10 (32.3)	
N1	75 (17.1)	18 (21.7)		28 (14.5)	2 (6.5)	
N2	91 (20.8)	12 (14.5)		38 (19.7)	5 (16.1)	
N3	132 (30.1)	20 (24.1)		57 (29.5)	14 (45.2)	
Pathologic TNM stage			0.07			0.58
I	64 (14.6)	12 (14.5)		29 (15.0)	5 (16.1)	
II	120 (27.3)	34 (41.0)		59 (30.6)	6 (19.4)	
III	230 (52.5)	32 (38.6)		98 (50.8)	18 (58.1)	
IV	24 (5.5)	5 (6.0)		7 (3.6)	2 (6.5)	
Pathologic Type			<0.01			
Intestinal	238 (54.3)	58 (69.9)		99 (51.3)	23 (74.2)	0.02
Diffuse	200 (44.7)	25 (30.1)		94 (48.7)	8 (25.8)	
Differentiation			0.44			0.91
Well-Moderate	109 (24.8)	24 (28.9)		58 (30.0)	9 (29.0)	
Poor	329 (75.1)	59 (77.8)		135 (70.0)	22 (71.0)	

Data are number of patients; data in parentheses are percentage unless otherwise indicated.

### Segmentation reproducibility

The Dice similarity coefficient of inter-observer segmentation was 0.91, which indicated a favorable agreement between readers.

### Radiomics feature extraction and signature establishment

**FIGURE 2:**
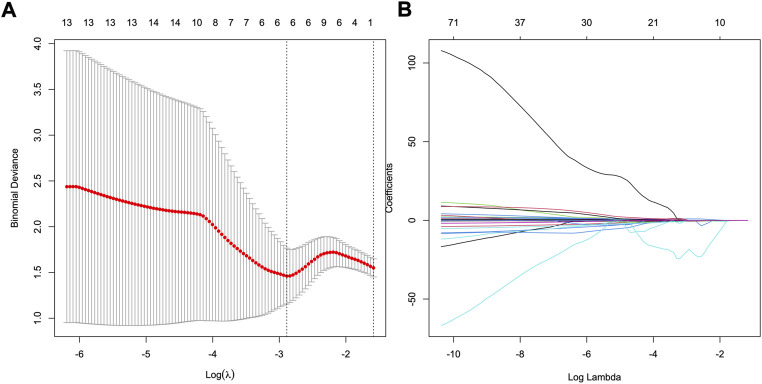
Feature selection using least absolute shrinkage and selection operator (LASSO) logistic regression. **(A)** Selection of tuning parameter (*λ*) in the LASSO model *via* 10-fold cross-testing based on minimum criteria. The AUC curve was plotted against log (*λ*). Dotted vertical lines were drawn at the optimal values by using the minimum criteria and the 1 standard error of the minimum criteria (the 1- standard error criteria). **(B)** LASSO coefficient profiles of the 96 selected features. A vertical line was plotted at the optimal *λ* value, which resulted in eight features with nonzero coefficients.

Of 2,577 features extracted from the VOIs of three phases images of the training set, 1836 features with intra- and interclass correlation coefficients <0.85 were excluded. Ninety-six significantly differentially changed features of the retained 741 features were identified between the HER2−and HER2+ groups and were brought into LASSO algorithm. Then, the radiomics signature was constructed based on 8 features with non-zero coefficients (Figure 2), include 3 features from AP, 4 features from PP, and 1 features from DP. The R-score calculation formula is presented in Supplementary formula. The distributions of R-scores are shown in Supplementary Figure S2.

### The evaluation of predictive performance of radiomics signature

**FIGURE 3:**
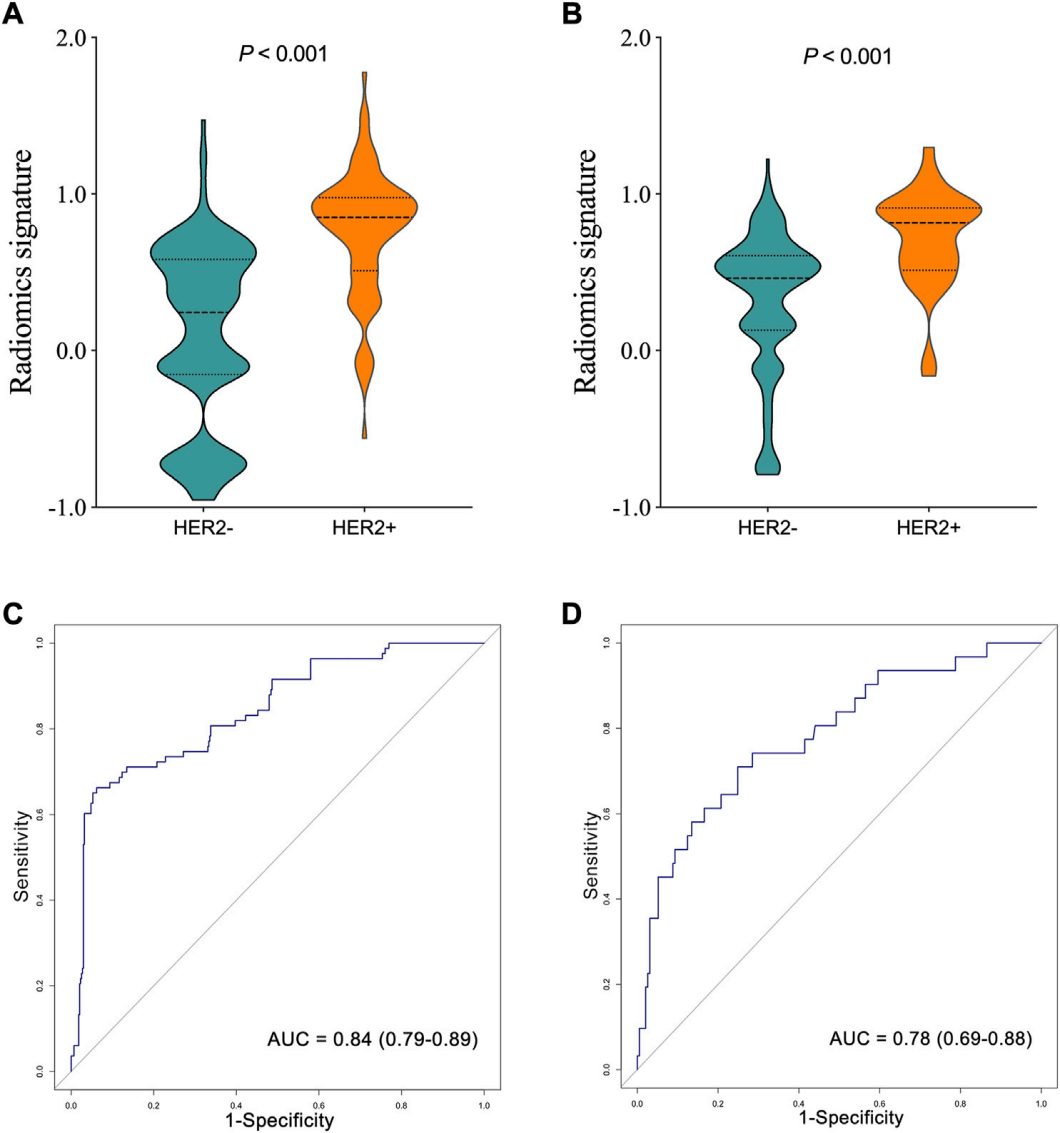
Comparison of radiomics score between human epidermal growth factor receptor 2 (HER2)—and HER2 + groups in the training **(A)**, and testing **(B)** sets. The ROC curves of the radiomics signature in the training **(C)**, and testing **(D)** sets.

The R-scores of HER2+ patients were significantly higher than those of HER2−patients in the training (*p* < 0.001, Figure 3A) and the testing sets (*p* < 0.001, Figure 3B). The radiomics signature demonstrated favorable performance in the training and the testing sets with AUCs of 0.84 (95% confidence interval (CI): 0.79–0.89, Figure 3C) and 0.78 (95% CI: 0.69–0.88, Figure 3D), respectively.

### Nomogram establishment and validation

**FIGURE 4:**
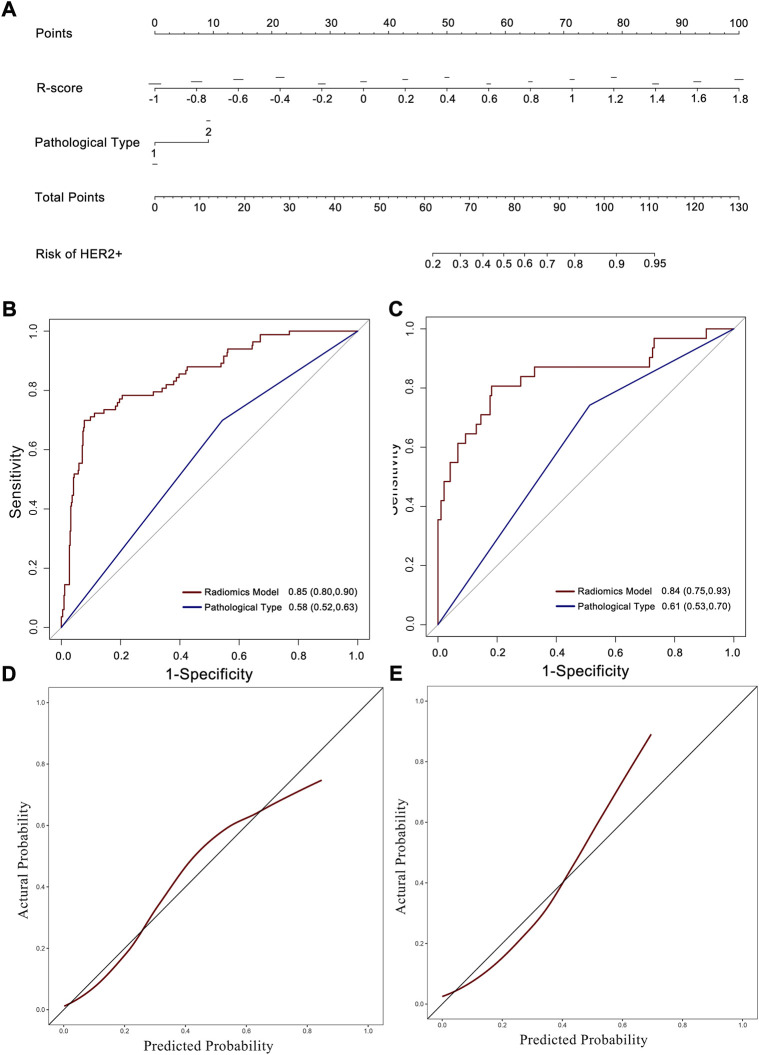
Nomogram developed with ROC and calibration curves. **(A)** A radiomics nomogram was developed in the training set with radiomics signature and pathological type incorporated. ROC curves of the radiomics nomogram and pathological type for the prediction of HER2 status in the training **(B)**, and testing **(C)** sets. Calibration curves of the nomogram in the training **(D)**, and testing **(E)** sets.

The uni- and multivariable logistic regression model were conducted to assess the association of HER2 status with R-score and the clinicopathological parameters in the training set. The radiomics signature and intestinal-type GC were demonstrated to be independent predictors for HER2 overexpression (Table 2). A nomogram was developed with the two factors (Figure 4A).

**TABLE 2:** Risk factors of HER2 overexpression in advanced gastric cancer.

Variable	Univariate logistic regression		Multivariate logistic regression	
	OR (95% CI)	*p* Value	OR (95% CI)	*p* Value
Gender	1.17 (0.75–1.80)	0.48		
AGE (<60 vs. ≥ 60)	0.86 (0.58–1.28)	0.45		
Tumor site	0.78 (0.84–1.24)	0.78		
TNM stage	0.94 (0.72–1.17)	0.48		
T stage	0.86 (0.69–1.07)	0.18		
N stage	0.94 (0.80–1.01)	0.42		
Pathological type	2.14 (1.39–3.31)	<0.01	1.92 (1.23–6.12)	0.02
Differentiation	0.88 (0.75–1.05)	0.12		
Radiomics signature	8.72 (4.25–18.41)	<0.01	6.13 (3.97–13.23)	<0.01

OR, odd ratio; CI, confidence interval.

The AUC of the nomogram in the training set was 0.85 (95% CI: 0.80–0.90; Figure 4B), which was higher than that of the radiomics signature and pathological type only. The testing set confirmed this result, with an AUC of 0.84 (95% CI: 0.75–0.93; Figure 4C). The calibration curve reflected a good fit for the nomogram in both the training (*p* = 0.71, Figure 4D) and the testing sets (*p* = 0.63, Figure 4E).

## Discussion

In the current study, we proposed a multi-phase CECT-based radiomics model incorporated with radiomics signature and pathological type as a noninvasive image biomarker for predicting HER2 overexpression in AGC patients. The model showed accurate discrimination power in both the training and testing sets. The radiomics signature may help clinician in the detection of patients with high risk of false results of HER2 overexpression due to tumor heterogeneity. In negative result from pathological examination, HER2 status need to be reevaluated if high-risk of HER2 overexpression suggested by radiomics model.

The ToGA trial firstly demonstrated that HER2+ AGC patients could benefit from trastuzumab treatment (Bang et al., 2010). After that, two phase II studies also showed that Trastuzumab could prolong the overall survival for Chinese HER2-positive AGC patients (Qiu et al., 2014; Gong et al., 2016). Based on these results, trastuzumab with chemotherapy is now the first-line treatment for patients with HER2+ AGC. However, compared to breast cancer, HER2 expression exhibits more heterogeneity in GC (Valtorta et al., 2015). The upregulation of HER2 induces the cell proliferation, migration, invasion and angiogenesis, which contribute to significantly increased heterogeneity in GC (Ciesielski et al., 2018). Accumulating evidence reveals that increased intratumoral heterogeneity of HER2 expression is associated with poor prognosis (Lee et al., 2013; Motoshima et al., 2018). For GC patients, the heterogeneity of HER2 status not only influences the accurate interpretation of HER2 status, but also associates with the treatment efficacy of anti-HER2 therapy (Wakatsuki et al., 2018; Yagi et al., 2019). The traditional biopsy-based assays maybe a barrier of personalized therapy as the detected gene amplification or mutations does not always reflect the full landscape of tumor cells. Therefore, there is still a need to find effective way of estimating clinical outcomes of HER2-targeted therapy.

The radiomics approach has been widely applied in the prediction of treatment efficacy in various types of tumors (Liu et al., 2019; Mattonen et al., 2019). Additionally, a growing body evidence demonstrates that the radiomics method can quantify the intratumoral heterogeneity. The high-dimensional radiomics features, which can hardly be visualized using human eyes, provide more details about intratumoral environment such as cell density, hypoxia, microvessel density (Ganeshan et al., 2012a; Ganeshan and Miles, 2013; Zhang et al., 2013). Moreover, the radiomics features extracted from the 3-dimensional lesions represent the entire landscape of tumor bulk (Mei et al., 2018). Previous study has revealed that texture features such as uniformity and entropy were correlated with worse survival of lung, esophageal, and head and neck squamous cell cancer (Ganeshan et al., 2012a; Ganeshan et al., 2012b; Zhang et al., 2013). Recently, Waugh et al. reported that CT texture features such as higher entropy were associated with increased intratumoral heterogeneity in HER2 positive breast cancer (Waugh et al., 2016). Sung et al.,2021 assessed the association of CT texture with outcome of GC patients received Trastuzumab treatment. However, this study only investigated the texture features extracted from the largest cross-sectional area of lesions (Yoon et al., 2016). In line with previous reports, we found that entropy was a crucial feature for prediction of HER2 status (Waugh et al., 2016). In addition, Grey Level Nonuniformity, which was selected in our signature, was suggested to be an important feature for measuring intratumoral heterogeneity (Aerts et al., 2014). These results revealed that the radiomics signature might provide insight into tumor heterogeneity and improved the explainability of our radiomics signature for prediction of HER2 status.

With the advancement of high-throughput sequencing techniques, several genomic classification systems, which reflecting the complicated genomic mechanisms underlying GC, have been proposed (Cancer Genome Atlas Research, 2014; Cristescu et al., 2015). The complicated alternation of signaling pathway induced by HER2 overexpression underlies the treatment efficacy of anti-HER2 therapy (Chen et al., 2012). Thus, further radiogenomics analysis which links radiomics features with genomic profile is warranted in future researches.

This study has some limitations. Firstly, although the radiomics analysis with volumetric features represented the status of the whole tumor bulk, bias may still be introduced as the test results of the specimens may not reflect the actual status of HER2 expression. Secondly, this retrospective study was conducted in a single center. Thirdly, due to the low positive rate of HER2 in GC in the Chinese population, our study only enrolled 114 HER+ patients Therefore, larger prospective multicenter studies are warranted to assess the generalizability of the radiomics signature.

In summary, we established and validated a multiphase CECT radiomics model which showed favorable prediction accuracy of HER2 in AGC patients and may improve confidence in clinical decision-making.

## Data Availability

The original contributions presented in the study are included in the article/Supplementary Material, further inquiries can be directed to the corresponding authors.
