# Supplementary material for: A multiphase contrast-enhanced CT radiomics model for prediction of human epidermal growth factor receptor 2 status in advanced gastric cancer
Source: Front Genet. 2022 Oct 7;13:968027. doi: 10.3389/fgene.2022.968027 (PMC9585247; doi:10.3389/fgene.2022.968027)
Supplement: Supplementary file 1 [file Table1.DOCX]

**Supplementary Data**

**I. Supplementary methods**

**CT scanning protocol**

Contrast-enhanced abdominal CT was performed for all patients using Somatom Sensation 64 (Siemens Medical Solutions, Forchheim, Germany) or a Discovery CT750 HD scanner (GE Medical Systems, Milwaukee, Wisconsin). Oral doses of water (500–1000 mL) were administered to distend the stomach before scanning. The scanning parameters were: tube voltage, 120 kVp; tube current, 150-200 mA; field of view, 350 mm × 350 mm; matrix, 512 × 512; and reconstruction section thickness, 1.25 mm. Arterial- and portal venous-phase scans were acquired after delays of 20 s and 60 s, respectively, following intra-phase injection of contrast medium (2.5 mL/s, 1.2 mL/kg; Omnipaque 300, GE Healthcare, Chicago, Illinois) via a syringe pump.

**Image Pre-Processing**

Before feature extraction, all CT images were resampled into 1.5× 1.5 × 1.5 mm^3^ resolution using linear interpolation. Z-score normalization was applied to unify intensity range across scanners.

**II Table S1. Radiomics feature type and associated Features**

| Feature Type | Methods | Feature name |
| --- | --- | --- |
| Shape-based |  | Maximum 3D diameter (M3D) |
|  |  | Maximum 2D diameter slice (M2DS) |
|  |  | Sphericity |
|  |  | Minor axis (MA) |
|  |  | Elongation |
|  |  | Surface volume ratio (SVR) |
|  |  | Volume |
|  |  | Major axis (MA1) |
|  |  | Surface area (SA) |
|  |  | Flatness |
|  |  | Least axis (LA)  Maximum 2D diameter column (M2DC) |
|  |  | Maximum 2D diameter row (M2DR) |
| First order-based | Histogram | Interquartile range (IQR) |
|  |  | Skewness |
|  |  | Uniformity |
|  |  | Median |
|  |  | Energy |
|  |  | Robust mean absolute deviation (RMAD) |
|  |  | Mean absolute deviation (MAD) |
|  |  | Total energy (TE) |
|  |  | Maximum |
|  |  | Root mean squared (RMS) |
|  |  | 90 Percentile |
|  |  | Minimum |
|  |  | Entropy |
|  |  | Range |
|  |  | Variance |
|  |  | 10 Percentile |
|  |  | Kurtosis |
|  |  | Mean |
| Texture-based | GLCM | Joint average (JA) |
|  |  | Sum average (SA) |
|  |  | Joint entropy (JE) |
|  |  | Cluster shade (CS)  Maximum probability (MP) |
|  |  | Idmn |
|  |  | Joint energy (JE) |
|  |  | Contrast |
|  |  | Difference entropy (DE) |
|  |  | Inverse variance (IV) |
|  |  | Difference variance (DV) |
|  |  | Idn |
|  |  | Idm |
|  |  | Correlation |
|  |  | Autocorrelation |
|  |  | Sum entropy (SE) |
|  |  | Sum squares (SS) |
|  |  | Cluster prominence (CP) |
|  |  | Imc2 |
|  |  | Difference average (DA) |
|  |  | Imc1 |
|  |  | Id |
|  |  | Cluster tendency (CT) |
|  | GLSZM | Gray level variance (GLV) |
|  |  | Zone variance (ZV) |
|  |  | Gray level non-uniformity normalized (GLNUN) |
|  |  | Size zone non-uniformity normalized (SZNUN) |
|  |  | Size zone non-uniformity (SZNU) |
|  |  | Gray level non-uniformity (GLNU) |
|  |  | Large area emphasis (LAE) |
|  |  | Small area high gray level emphasis (SAHGLE) |
|  |  | zone percentage (ZP) |
|  |  | Large area low gray level emphasis (LALGLE) |
|  |  | Large area high gray level emphasis (LAHGLE) |
|  |  | High gray level zone emphasis (HGLZE) |
|  |  | Small area emphasis (SAE) |
|  |  | Low gray level zone emphasis (LGLZE) |
|  |  | Zone entropy (ZE) |
|  |  | Small area low gray level emphasis (SALGLE) |
|  | GLRLM | Short run low gray level emphasis (SRLGLE) |
|  |  | Gray level variance (GLV) |
|  |  | Low gray level run emphasis (LGLRE) |
|  |  | Gray level non-uniformity normalized (GLNUN) |
|  |  | Run variance (RV) |
|  |  | Gray level non-uniformity (GLNU) |
|  |  | Long run emphasis (LRE) |
|  |  | Short run high gray level emphasis (SRHGLE) |
|  |  | Run length non-uniformity (RLNU) |
|  |  | Short run emphasis (SRE) |
|  |  | Long run high gray level emphasis (LRHGLE) |
|  |  | Run percentage (RP) |
|  |  | Long run low gray level emphasis (LRLGLE) |
|  |  | Run entropy (RE) |
|  |  | High gray Level run emphasis (HGLRE) |
|  |  | Run length non-uniformity normalized (RLNUN) |
|  | NGTDM | Coarseness |
|  |  | Complexity |
|  |  | Strength |
|  |  | Contrast |
|  |  | Busyness |
|  | GLDM | Gray level variance (GLV) |
|  |  | High gray level emphasis (HGLE) |
|  |  | Dependence entropy (DE) |
|  |  | Dependence non-uniformity (DNU) |
|  |  | Gray level non-uniformity (GLNU) |
|  |  | Small dependence emphasis (SDE) |
|  |  | Small dependence high gray level emphasis (SDHGLE) |
|  |  | Dependence non-uniformity normalized (DNUN) |
|  |  | Large dependence emphasis (LDE) |
|  |  | Large dependence low gray level emphasis (LDLGLE) |
|  |  | Dependence variance (DV) |
|  |  | Large dependence high gray gevel emphasis (LDHGLE) |
|  |  | Small dependence low gray level emphasis (SDLGLE) |
|  |  | Low gray level emphasis (LGLE) |
| Wavelet-based | First-order statistic and texture of wavelet decomposition.  Decomposition levels: LLL, LLH, LHL, LHH, HLL, HLH, HHL, HHH. | First-order features |
|  |  | GLCM features |
|  |  | GLSZM features |
|  |  | GLRLM features |
|  |  | GLDM features |

GLCM, [gray-level co-occurrence matrix], describe the second-order joint probability function of the voxel intensities within the contoured volume; GLSZM, [gray-level size-zone matrix], quantify the number of connected voxels within the contoured volume that share the same gray level intensity; GLRLM, [gray-level run-Length matrix], quantify the number of consecutive voxels that have the same gray level value; NGTDM, [neighboring gray-tone difference matrix], quantify the difference between a gray value and the average gray value of its neighbors within 3×3×3 voxels neighborhood window; GLDM, [gray-level dependence matrix], quantify the gray level dependencies in the contoured volume which is defined as the number of connected voxels within a specific distance that are dependent on the center voxel; Decomposition levels, i.e. LLH interpreted as the high-pass sub band, resulting from directional filtering of the volume with a low-pass filter along x-direction, a low pas filter along y-direction and a high-pass filter along z-direction

**III Radiomics signature formula**

Radiomics score = 1.533 × PP-GLRLM.Gray Level NonUniformity Normalized

-0.225 × PP-GLSZM.Size Zone Non-Uniformity Normalized

+1.032×AP-Wavelet-LLL-Firstorder.Entropy

+0.009 ×AP-GLSZM.Gray Level Variance

-2.123 × PP-Wavelet-HHH-Firstorder.Energy

-0.933 × PP-Wavelet-LHH-Firstorder.Skewness

+0.377 × AP-NGTDM.Complexity

+0.255 ×DP-Maximum 3D diameter

**IV. Supplementary Figures**

**Figure S1 Recruitment pathways for patients**

**
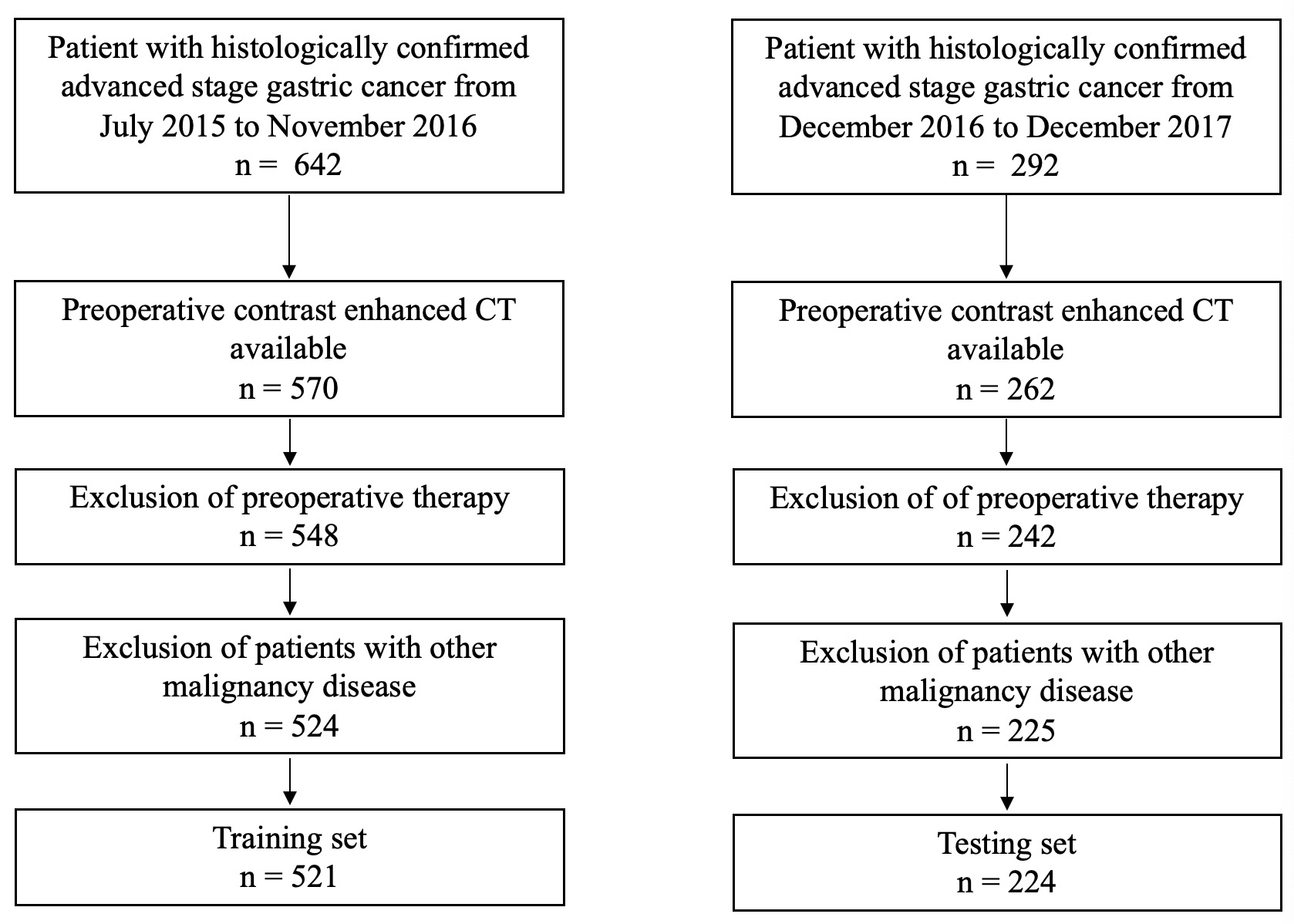
**

**Figure S2 Radiomics score for each patient in the entire cohort**

**
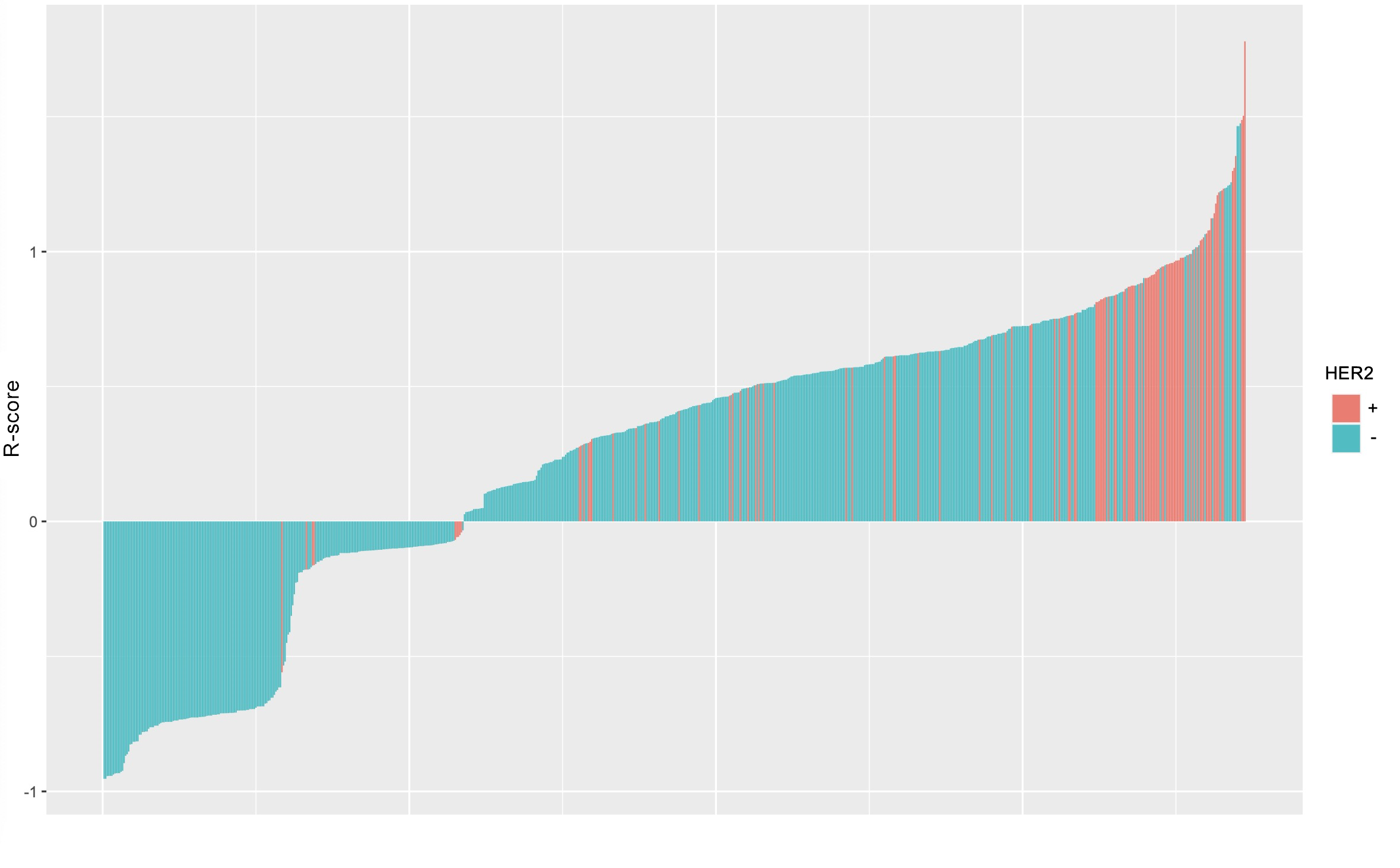
**
